# Self-monitoring of blood pressure and self-assessment of self-care: an interview study among patients with hypertension

**DOI:** 10.1186/s12875-026-03269-7

**Published:** 2026-03-14

**Authors:** Elnura Halmambetova, Evalill Nilsson, Cecilia Fagerström, Kristofer Årestedt, Mimmi Bjöersdorff, Linda Ljungholm

**Affiliations:** 1 Department of Research, , Region Kalmar County, Kalmar, Sweden; 2 Department of Medicine and Optometry, Faculty of Health and Life Sciences, Linnaeus University, Kalmar-Växjö, Sweden; 3 Department of Health and Caring Sciences, Faculty of Health and Life Sciences, Linnaeus University, Kalmar-Växjö, Sweden; 4 Qulturum – Center for Learning and Innovation, Region Jönköping County, Jönköping, Sweden

**Keywords:** Digital health, E-heath, Home monitoring, Hypertension, Patient-reported data, Primary care, Self-care, Self-monitoring, Thematic analysis

## Abstract

**Background:**

Managing and controlling hypertension remains a challenge for many patients, underscoring the need for more effective strategies. Although self-monitoring of blood pressure can greatly support the management of hypertension, combining it with questionnaires for self-assessment of self-care might further improve blood pressure control and patients’ health situation. There is extensive research on self-monitoring and self-management but research on the presumed added value of using self-assessment questionnaires in conjunction with self-monitoring of blood pressure, and how patients perceive these methods, remains lacking.

**Methods:**

Qualitative explorative study design involving 15 semi-structured in-depth interviews with primary care patients with hypertension who self-monitored their blood pressure and who were given a self-assessment questionnaire on self-care of hypertension for review. Data were analysed inductively using reflexive thematic analysis.

**Results:**

Self-monitoring of blood pressure promoted increased understanding of one’s body and the self-assessment questionnaire regarding self-care elicited reflection on one’s health situation. This was seen as providing opportunities for patients to actively co-create health and self-care in collaboration with healthcare professionals. The latter were expected to become more engaged and better meet patients’ information needs about the illness and treatment, and to make use of patients’ self-reported data for shared decision-making in hypertension management. Although sustainable self-care is often portrayed as autonomous, it appeared to rely on active support and engagement from healthcare professionals.

**Conclusions:**

Self-monitoring of blood pressure combined with self-assessment of self-care was thought to have the potential to promote deeper awareness of personal health and improve the patient-professional relationship. This highlights that sustainable self-care requires quality support and meaningful engagement between patient and healthcare professional, rather than being a completely independent endeavour. Further research could shed light on whether self-monitoring of blood pressure combined with consistent use of self-assessment questionnaires can promote a culture of collaboration and shared decision-making in hypertension care.

**Supplementary Information:**

The online version contains supplementary material available at 10.1186/s12875-026-03269-7.

## Background

 Hypertension is the most common risk factor related to the development of severe cardiovascular diseases such as myocardial infarction and stroke [1]. Despite the risks, undertreatment of hypertension is often reported in the literature [2]. Many people go undiagnosed, and even among those who are treated, about half do not reach target blood pressure values [3, 4]. These results underscore the global challenge of hypertension and the need for effective strategies to improve awareness, treatment, and control of hypertension [5].

Self-monitoring of blood pressure has become an essential strategy in effective management of hypertension [6]. It can be described as recognizing symptoms and measuring blood pressure to evaluate health [7, 8]. Self-monitoring of blood pressure can enhance patient-provider interactions [9], increase disease knowledge [10], strengthen motivation for lifestyle changes, improve medication adherence, and promote self-care [11] and self-management [11, 12]. When combined with self-care education, it can lower blood pressure—even in resistant hypertension—without increasing healthcare utilization or the frequency of adverse events [13, 14].

Although self-monitoring of blood pressure can provide an increased sense of security, some patients may feel burdened by the technology [15], especially if they have difficulty understanding blood pressure [16]. Hence, tailored training and support are essential to maintain accuracy when implementing self-monitoring, especially among patients with low health literacy [17].

Self-assessments of health and self-care through questionnaires have been shown to improve patient-related outcomes in general [18], and can potentially complement self-monitoring of health in several ways. Whereas self-monitoring of blood pressure can provide quantitative biomedical data, self-assessed questionnaires can contribute with data about a patient’s perceived health situation. If a patient reports increased fatigue alongside rising blood pressure, their healthcare provider can adjust the treatment plan accordingly. Such integration recognizes that although traditional clinical metrics such as blood pressure values are important, there is also a need to look at how disease and treatment affect a patient’s self-reported health status and self-care, offering a more holistic view of the patient’s health situation [19].

Self-reported blood pressure can be seen as a relatively insensitive measure for symptom changes and health experience, meaning that supplementing such measurements with self-assessments of self-care could potentially contribute to better blood pressure management. However, although there is extensive research advocating for self-monitoring of blood pressure, research on utilization of self-assessment of self-care in relation to self-monitoring is limited. Specifically, it is unknown how patients perceive these practices. Hence, exploring patients’ perspectives is crucial for understanding whether they see any value in engaging with self-monitoring of blood pressure and self-assessment of self-care. This is particularly important as patients are the end users of healthcare services and can shape their own health outcomes through participation in treatment decisions and adherence to medical recommendations. Therefore, this study aimed to explore experiences of self-monitoring of blood pressure, and the potential value of using self-assessment of self-care in addition to self-monitoring among patients with hypertension.

## Method

### Design

This study employed an explorative qualitative research design with individual interviews to provide deeper insight into patients’ experiences of self-monitoring of blood pressure, and their perspectives on the potential use of self-assessment of self-care in relation to self-monitoring. Reflexive thematic analysis was used to identify, analyse, and report patterns (themes) within the interview data [20].

### Setting

The study was carried out in south-eastern Sweden, where work is underway to establish self-monitoring of health in patients’ home environment, including for patients with hypertension who are managed in primary care.

Although self-monitoring routines may differ slightly between primary healthcare centres (PHCCs), patients with hypertension usually either receive a blood pressure cuff from their PHCC or buy an approved cuff at a pharmacy, which may be calibrated by healthcare professionals at the PHCC. Patients are expected to self-measure their blood pressure after receiving instructions from their PHCC and then report values back to the PHCC regularly, at agreed intervals. Many PHCCs across Sweden offer patients use of a so-called ‘blood pressure corner’ or ‘blood pressure room’, a secluded, quiet place at the PHCC where they can self-measure their blood pressure using the PHCC’s equipment.

Patients are usually asked to self-monitor their blood pressure in conjunction with medication adjustments, lifestyle changes, and before their regular check-ups. The self-monitored blood pressure values may be reported digitally via an app, via a message function in the national healthcare portal 1177, by e-mail, or in writing on paper or a preprinted form from the PHCC. Follow-up of self-monitoring usually takes place when blood pressure values fall outside the normal range.

### Participants

Patients were recruited from PHCCs and from the county’s resident panel, where residents (including patients) can register to share their experiences and insights to support research initiatives aimed at enhancing the quality of public service delivery, particularly within the healthcare sector.

Purposeful sampling was used with the following inclusion criteria: patients with a hypertension diagnosis, who have the cognitive and linguistic ability to complete an interview and read a questionnaire. To ensure variation in the sample, the study aimed to include both patients with newly diagnosed and those with well-established hypertension, with a mixture of male and female patients of different ages.

**Fig. 1:**
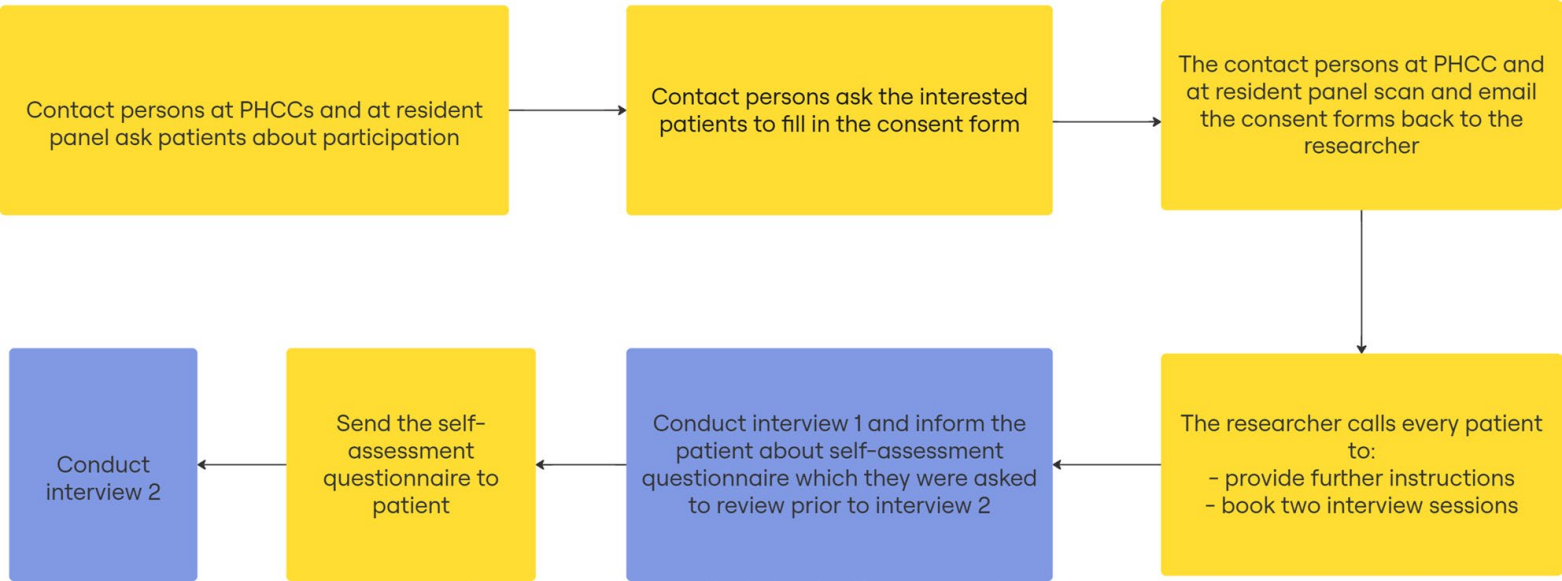
Patient recruitment flowchart

Initially, the research group contacted the resident panel’s contact person as well as nurses who worked with blood pressure patients at each PHCC. The contact persons then initiated contact with the patients and forwarded relevant contact information to the research group (Fig. 1). The first round of interviews was conducted between December 2023 and March 2024, and the second round during May and June 2024.

A total of fifteen patients were interviewed, ten of whom were female (Table 1). The age of the participants ranged between 38 and 81 years, with a median age of 68 years. The duration of the hypertension diagnosis ranged from 3 months up to 25 years, with a median of 5.5 years. Data on demographic characteristics and the presence of comorbidity were collected. Participants’ other medical diagnoses included asthma, sleep apnoea, cancer, type 2 diabetes, and neuropsychiatric disabilities.

**Table 1:** Demographic characteristics of the study population

Interview 1	Interview 2	Sex	Age (years)	Years with hypertension
X	X	M	65–80	˂5
X	X	F	65–80	5–10
X		M	< 65	5–10
X	X	M	65–80	˃10
X	X	F	65–80	˂5
X	X	M	65–80	˂5
X		F	65–80	˃10
X	X	F	65–80	˃10
X	X	F	˃80	˂5
X	X	F	< 65	˂5
X	X	F	65–80	5–10
X		F	< 65	˂5
	X	M	65–80	˂5
	X	F	˃80	˃10
	X	F	65–80	˂5

### Data collection

The data collection was conducted in two interview rounds, each based on a separate semi-structured interview guide. The reason for conducting two separate rounds of interviews was that self-monitoring of blood pressure and self-assessments of health and self-care were originally intended to be the focus of two separate studies. However, during the data analysis process, it became clear that there was significant overlap and connection between the results of the two interview rounds. This realization led us to merge the studies into one study with a broader purpose, allowing for a more integrated understanding of the participants’ experiences while ensuring in-depth data collection.

The first interview round focused on patients’ experiences of self-monitoring of their blood pressure. This interview guide also contained questions about patients’ perceptions of health when living with hypertension. The interviews in the first round lasted from 17 min to 42 min, with a median length of 32 min.

The second interview round focused on capturing patients’ thoughts about the potential use of self-assessment questionnaires in addition to self-monitoring of blood pressure. The questionnaire in the present study included items focused specifically on self-care related to hypertension [21]. The participants in the second round of interviews received the questionnaire several days before their interview was carried out, so they could read it and familiarize themselves with it prior to the interview. During the interviews, the patients were asked for their thoughts about the potential use of such questionnaires, in addition to self-monitoring of blood pressure (i.e., they had no personal experience of answering such questionnaires in a clinical situation). The interviews in the second round lasted from 18 min to 52 min, with a median length of 27 min. All interviews were recorded and transcribed verbatim.

### Data analysis

The analysis was carried out in six steps using Braun’s and Clarke’s reflexive thematic analysis [20]. Data analysis started with reading through all the data material while noting thoughts and ideas relevant to the purpose of the study. In the second step, meaning units were identified in the text and assigned with codes. The codes were developed inductively, meaning that the analysis had a descriptive and exploratory orientation. This step was carried out by three of the researchers, all with different disciplinary backgrounds. In the third step, the codes were assigned to code labels, corresponding to the development of initial themes. Through mind mapping, data were organized visually, with relationships between codes and initial themes being determined. In step four, the initial themes were reviewed through revision against the full dataset. In step five, the themes were refined and defined. At this stage, the subthemes were used to capture and highlight important aspects of the themes, helping to outline the scope, boundaries, and core concepts of the themes. The subthemes provided a more detailed and descriptive analysis close to the data, whereas the themes were at a more interpretative level. Next, the themes were reviewed to ensure that they accurately captured the essence of the data in relation to the study aim. The generation of themes was conducted by five of the researchers involved in the project. The essence of each theme was given a name. Lastly, in step six, the analytical process was completed through writing and finalizing the analytical report [20]. At this stage, the themes were further refined through going back and forth between the original data and the themes. Patient quotations were used to illustrate that the results reflected their experiences and perspectives. An example of this analytical process is provided in Appendix A.

## Results

**Fig. 2:**
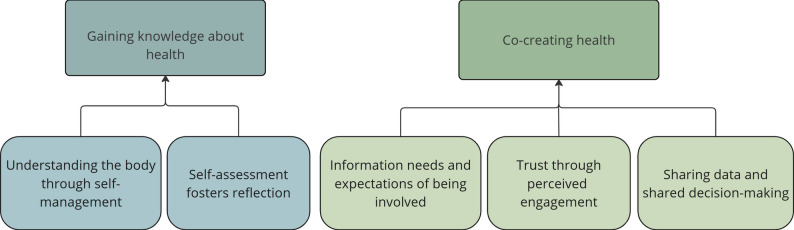
Patients’ experiences of self-monitoring of blood pressure and their perspectives on the use of self-assessment of self-care

Two themes were developed to capture patients’ experiences of self-monitoring of blood pressure and their thoughts about using self-assessment questionnaires regarding self-care in addition to self-monitoring. The themes were called *Gaining knowledge about health* and *Co-creating health*, and contained two and three subthemes, respectively (Fig. 2).

The theme *Gaining knowledge about health* reflects the effect of self-monitoring and self-assessment on the individual patient, whereas *Co-creating health* emphasizes the central role of patient-professional interaction in hypertension care (Table 2).

**Table 2:** Overview of themes, subthemes, and their characteristics

Themes (bold) and subthemes	Characteristics
**Gaining knowledge about health**	A process where the practices of self-monitoring and self-assessment help patients to gain knowledge about their health
Understanding the body through self-management	A process of understanding the body through self-monitoring, interpreting, and managing blood pressure values
Self-assessment fosters reflection	A process of reflecting, evaluating, and making sense of one’s own health situation through self-assessment of self-care
**Co-creating health**	A collaborative process where patients and healthcare professionals actively exchange information to support self-care
Information needs and expectations of being involved	Co-creating health can be facilitated through addressing patients’ information needs and expectations, which are crucial in sustainable self-care
Trust through perceived engagement	Co-creating health can be facilitated through the collection of self-assessed self-care data, which fosters patients’ trust in healthcare professionals
Sharing data and shared decision-making	Co-creating health can be facilitated through sharing data on self-monitored blood pressure values and self-assessed self-care, which supports shared decision-making in self-care

### Gaining knowledge about health

This theme captures how self-monitoring of blood pressure and self-assessment of self-care can contribute to gaining knowledge about one’s own health. The practice of self-monitoring of blood pressure facilitated understanding of the body through self-management of hypertension, whereas the use of a questionnaire to self-assess self-care was described as a potential opportunity to reflect on one’s own health situation.

### Understanding the body through self-management

Self-monitoring of blood pressure was described as increasing participation in one’s own care, which facilitated gaining knowledge about one’s health through understanding of one’s own body. Patients measured their blood pressure prior to scheduled contact with healthcare, if they felt there was something off with their wellbeing, or if they felt symptoms of hypertension. Symptom recognition was at times challenging, partly due to difficulties noticing the symptoms and partly due to confusion of symptoms related to other diagnoses. If an increase in blood pressure was recognized, this was based on symptoms such as headache, dizziness, ringing in the ears, and heart palpitations. On some occasions, these symptoms were accompanied by psychological discomfort such as feelings of exhaustion, grumpiness, stress, or being rushed.

According to the patients, symptom recognition facilitated the maintenance of normotension through self-care strategies such as drinking water or going for a walk, thereby helping them to self-manage their hypertension.

If my blood pressure is high, then I rest and take things a little easier, and measure it again. It usually decreases after a while. (R10)

Although self-monitoring could help patients understand their body, patients noted that the practice of self-monitoring also could have side effects. Awareness of high blood pressure could make some patients stressed and anxious, as the first thing they thought of in that situation was the risk of stroke.

When the blood pressure rises, I might get a little stressed and then it might get even higher. (R4)

Although stress and anxiety were relatively common when patients had recently started self-monitoring their blood pressure, monitoring at later stages had the potential to provide reassurance and peace of mind. This was mostly due to patients’ learned ability to mitigate blood pressure fluctuations through practices such as relaxing and performing measurements in a quiet environment.

### Self-assessment fosters reflection

The use of self-assessments of self-care in hypertension care was considered to potentially help patients gain knowledge about their health by promoting reflection on their health situation. This was described as valuable, especially if questionnaires would be sent out along with each invitation to a booked healthcare visit. According to the patients, filling out the questionnaire at home in a calm environment would provide an opportunity to reflect on their health and self-care. The self-assessment instrument on self-care used in the present study included questions about lifestyle and patients described it as a potential reminder to take care of oneself, raising their awareness about how they lived their life and providing food for thought. Thus, according to patients, self-assessments could support them in developing a more comprehensive understanding of their personal health status, while counteracting tendencies toward habitual or ineffective health management practices.

I noticed that my blood pressure got better when I lost weight, so I became aware that if I gain weight, my blood pressure also increases. This knowledge gives me a lot of security and awareness […] The self-assessment questionnaire together with the self-monitoring of blood pressure could contribute to better health, because I become aware that I feel better if I lose weight. (R6)

As patients felt they could not always rely on their memory, they suggested that filling out questionnaires regularly could be valuable to track changes in their health and self-care over time.

### Co-creating health

This theme captures the information needs from and expectations on hypertension care expressed by patients. When the needs were recognized and met, patients reported feeling more comfortable engaging in self-monitoring and self-care. Collecting questionnaire data on self-assessed self-care was reported to promote patients’ trust in healthcare professionals. Sharing self-reported blood pressure values along with self-assessed self-care data was described as facilitating shared decision-making through increased patient participation.

### Information needs and expectations of being involved

Patients’ experiences of hypertension care and self-monitoring of blood pressure underscored a need for more information and education about the disease and care processes. Highlighting the notion that ‘my body should not be a secret to me’, patients expressed a desire for greater involvement in information sharing to support their health and self-care efforts. Information leaflets on hypertension and its management, including self-care activities, written in lay terms, were described as a good way to get informed.

Patients perceived ambiguities regarding the care process; clear hypertension care plans were lacking. Although the easily accessible electronic health records were described as a good source of information, not all patients were aware of their availability. Patients noted that the fact that the drug treatment was likely to be life-long created anxiety, especially when a drug prescription was not followed up in relation to wellbeing.

I wish someone would contact me when the prescriptions need to be renewed, that I was at least asked how I am doing, and not just handed a new prescription… I feel like no one cares. (R5)

Patients expressed a strong need for information and feedback about the treatment, especially when changing the medication or adjusting the dose. Information about side effects and possible interactions was also desired, particularly among patients who had multiple diseases and medications. Such information was seen as important for enabling effective self-monitoring and self-care.

Although most patients made a clear connection between stress and high blood pressure, the lifestyle support from healthcare professionals was described as minimal. Some patients expressed a fear of exercising and felt that lifestyle support in the form of tailored guidance would have been helpful. Patients stated that it was difficult for them to determine if exercise-induced nausea or shortness of breath indicated potential harm. Patients suggested that their need for information and education could be met through more committed and closer healthcare contacts, which according to them could be facilitated through digital communication methods. Lastly, meeting with different healthcare professionals meant that patients received different information about their blood pressure target values, making self-monitoring more difficult. This led patients to seek help on the internet, though it was difficult for them to determine which information was relevant to their unique situation. Therefore, they expressed a need for consistency and continuity in care, which they found to be lacking when they met several different physicians instead of a named primary care contact (e.g., accountable physician). Patients expected their physician to be well-acquainted with their individual circumstances, adopt a service-oriented approach, and actively involve them by providing detailed information about their care plan. Further, they expected the physician to inquire about their well-being, explain and discuss issues of personal relevance, and monitor their self-care practices, thereby ensuring that they did not feel solely responsible for their self-monitoring.

I think an understanding healthcare professional is the most important thing in general for healthcare, that they can explain things and how things work. (R12)

Patients believed that understanding healthcare professionals were not only aware of how high blood pressure could affect a patient’s daily life and work but also had an understanding of the broader life consequences associated with a hypertension diagnosis.

### Trust through perceived engagement

This subtheme captures patients’ experiences of living with hypertension, which meant living with a fear of severe sequelae such as stroke and being aware of a higher risk of developing cardiovascular diseases despite not always feeling any symptoms. However, patients stated that hypertension care lacked sufficient support from healthcare professionals. In comparison to the care of the patients’ other chronic diseases, the provision of hypertension care was described as less proactive. Although there were frequent check-ups during the early phase of the medical investigation, the follow-up in the later stages of the care process was described as almost non-existent, meaning that hypertension care after the initial phase was based solely on the patient’s initiative and self-assessments. Some patients perceived healthcare as rejecting and unwelcoming, which contributed to a feeling of being a burden. Such experiences could leave them feeling lost or excluded and undermined their trust in the healthcare system. According to the patients, the use of self-assessment questionnaires on self-care could make the patients feel like ‘they care about me’ and indicate healthcare professionals’ interest in the patients’ health and disease management, fostering a sense of security and helping to establish trust in healthcare professionals.

[If I am asked to answer this kind of questionnaire] I feel that they care, and that they have a competent way of working, and are up to date and innovative. (R6)

Furthermore, patients perceived primary care as overburdened, which they believed limited healthcare professionals’ ability to focus on the needs of each individual patient. However, the use of self-assessment questionnaires on self-care was endorsed by patients, both as a way for healthcare professionals to identify the individuals who need the most support, and as a way for patients to get more personalized care.

I can imagine that it is not easy for them either to know what all their patients think and like […] Questionnaires could be valuable, so that not everyone is treated in the same way, because everyone has a different way of thinking, and feels differently. (R1)

Moreover, it was expressed that the use of self-assessment questionnaires could potentially even out differences between healthcare professionals in hypertension care. The questionnaires could serve as a checklist for healthcare professionals in the provision of care. This was described in relation to patients’ differing care experiences with different healthcare professionals. Patients believed that a feeling of security could also be facilitated by healthcare professionals’ use of such questionnaires, as it would enable healthcare professionals to keep a closer eye on patients. If something were to happen, continuous monitoring of health and self-care would provide an opportunity for retrospection and provide insights on when and why changes occurred.

### Sharing data and shared decision-making

The patients stated that self-monitoring of blood pressure led them to feel more involved in their own care. According to their narratives, sharing data on self-assessed self-care along with self-reported blood pressure values might further increase their participation and self-care, but only if healthcare professionals were to review and provide feedback on the shared data. Patients suggested that recording blood pressure values and receiving feedback in a shared digital system would be a good way to jointly monitor blood pressure.

My brother lived in northern Sweden, where they had a blood pressure machine at the PHCC that patients could use on their own. If something was out of the ordinary with your blood pressure, a signal would go to the computer and then to the doctor. (R10)

Furthermore, patients stated that answering a question in the written form could be more valuable than receiving the same question during a quick clinical encounter. Answering a question in writing required actively considering their health situation and created an opportunity to give specific information on their health, which could help healthcare professionals to better understand and support individual patients in self-care. Patients stated that the questionnaire could form a valuable basis for discussion, facilitating effective communication around patient´s needs. Therefore, they wished that healthcare professionals would familiarize themselves with a patient’s answers before each clinical encounter, so that discussion of the results would be worthwhile, for example in evaluation of medication, diet, or exercise interventions. Furthermore, joint discussion of self-assessed self-care could increase a patient’s sense of responsibility for managing their hypertension. However, for this to be realized, patients emphasized that they need to be informed about how to manage hypertension.

It is also my responsibility to manage my health. But I need healthcare professionals who I can talk to about how best to manage it. (R15)

## Discussion

To our knowledge, this is one of few studies exploring patients’ experiences of self-monitoring of blood pressure and the potential value of using self-assessment of self-care in hypertension care in conjunction with self-monitoring. The results suggest that the practices of self-monitoring and self-assessment of self-care could help patients gain knowledge about their health, which could provide a way for them to actively co-create health and self-care in collaboration with healthcare professionals. Sharing data on self-assessed self-care and self-measured blood pressure values could further promote patients’ health and self-care through shared decision-making.

Understanding of one’s body by means of self-monitoring of blood pressure can be explained based on interoception, which is awareness of internal bodily signals [22]. Interoception plays a significant role in symptom recognition and is seen as a crucial link between self-care monitoring (observing oneself for symptoms) and self-care management (taking actions to manage symptoms). This suggests that improving symptom recognition for elevated blood pressure could enhance patients’ self-care practices. Hence, to optimize the practices of self-monitoring and self-care, healthcare professionals might support patients in symptom recognition through developing their awareness of internal body signals. According to the patients in the present study, this could also be facilitated by the use of self-assessment questionnaires, which were considered to foster self-reflection and help patients follow changes in their health and self-care over time.

Addressing patients’ information needs and expectations is essential for achieving patient-centred care in hypertension, as self-care activation requires knowledge of one’s disease and support in symptom interpretation [9]. This means that sustainable self-care can be achieved through active support from healthcare professionals. Although technical devices for self-monitoring of blood pressure can be essential means for evaluating health, they are not sufficient on their own. Self-monitoring of blood pressure and self-care efforts over time should complement, rather than replace, the ongoing patient-professional relationship [9]. Therefore, healthcare professionals’ engagement in patients’ health situations through shared data is crucial for effective self-care. As also shown in the present study, the absence of such support may leave patients with feelings of being alone in managing their condition, struggling due to limited knowledge, and being dissatisfied with care due to perceiving healthcare as impersonal, inconsistent, and poorly coordinated [23].

Furthermore, patients in the present study indicated that the use of self-assessment of self-care could foster trust in healthcare professionals and might help patients articulate their health needs more effectively, thereby increasing their participation in their own care [24]. Self-monitoring of blood pressure, symptoms, and self-care, combined with the provision of tailored information based on self-assessed care, can also foster mutual understanding between patients and healthcare professionals, which in turn can give patients the experience of more coherent and continuous care [25].

The present study suggests that self-monitoring of blood pressure and self-assessment of self-care may also promote shared decision-making in self-care. This is supported by studies indicating that the use of self-assessment questionnaires as decision aids can improve health outcomes and enhance the quality of decision-making [18]. In line with what the patients in the present study stated, it has been shown that patients’ understanding of high blood pressure and the interest a clinician shows for a patient can facilitate shared decision-making, while a perceived lack of compassion, relationship hierarchies, and time constraints can act as barriers [26].

In the present study, co-creation of health was considered to involve patient participation and shared decision-making, which were enabled by the self-assessment questionnaire and self-monitoring as tools to engage patients in their own health and self-care. These findings support the theory that the relationship between patient participation and shared decision-making is mainly built on patient engagement [27]. This suggests that rather than uniformly promoting shared decision-making for all patients, healthcare professionals should aim to promote patient engagement first and primarily engage patients with relatively high participation in shared decision-making.

Although self-care is often portrayed as an independent and autonomous activity, our findings suggest that its sustainability is closely linked to active support and engagement from healthcare professionals. The theme of ‘Co-creation of health’ reflects this interdependence and indicates that patients benefit from collaborative relationships in which healthcare professionals provide tailored information, guidance, and patient education to improve health literacy. Such interactions may not only facilitate informed decision-making but may also strengthen patients’ confidence and ability to engage in self-care monitoring, which is defined as an activity that requires paying attention, confidence, and routines for tracking symptoms, signs, and actions [8]. This desire for support and engagement may be partly explained by the characteristics of our sample – it was predominantly composed of older women, a demographic group that may be vulnerable and more dependent on professional support. Nevertheless, this finding suggests that sustainable self-care requires quality support and meaningful engagement between patients and healthcare professionals, rather than operating as a completely independent endeavour. Although the present study suggests that self-assessment of self-care can help engage patients in their care by encouraging them to reflect on their health and self-care, thereby improving engagement and adherence to self-monitoring, it can be challenging to maintain sustained patient engagement in both self-monitoring and self-assessment [21]. Integrating patient-reported data into existing clinical workflows can also be challenging as healthcare providers need systems that can seamlessly integrate those data into their routine practice without causing disruption [28]. Such systems are generally lacking today, at least in Sweden. This highlights the need for more research on effective integration strategies, including the development of user-friendly digital tools, workflow adaptations, and organizational support to ensure that data are both accessible and actionable in clinical decision-making.

### Strengths and limitations

The credibility of this study was enhanced through researcher triangulation, as the researchers formed a multidisciplinary group. To minimize researcher bias, the researchers continuously discussed and reflected on their pre-understandings throughout the analysis process. Due to time constraints, the participants were not involved in the analysis process to verify their experiences. Furthermore, patients may not always be able to judge the correctness of the analysis at an aggregated level, which might threaten confirmability [28, 29].

Although the guidelines for the work process for high blood pressure care are relatively uniform across Sweden, the self-monitoring process currently implemented may differ between regions, and in relation to other countries. This can affect the transferability of the study. To increase transferability, the participants and the context have been described carefully. However, transferability should always be considered by the reader [30].

All interviews were conducted by the same person, which can be seen as a strength due to consistency. A potential weakness of the study might be that interview studies always entail a certain amount of subjectivity. However, within reflexive thematic analysis, subjectivity is seen as something valuable rather than problematic, as reflexive research treats knowledge as situated, meaning that it inevitably and inescapably includes the researcher’s practices of knowledge production. Hence, there is always a risk of subjectivity that can affect confirmability [20], as reflexive thematic analysis is based on the researcher’s interpretations. Nevertheless, by maintaining a transparent account of the data and the analysis process, the results can be verified [31]. In line with Braun’s and Clarke’s conceptualization of reflexive thematic analysis, the study aimed for sufficient data richness rather than saturation in the traditional sense. In reflexive thematic analysis, *data saturation* is not always defined as ‘no new codes emerging’, as the process is iterative, interpretive, and influenced by the researcher’s reflexivity [32]. The sample is considered adequate once the data provide the depth and diversity of perspectives necessary to construct meaningful and well-supported themes. Although additional interviews might have introduced further nuance, they were not likely to fundamentally alter the central themes, which were well-evidenced across the data set. Lastly, according to the information power framework described by Malterud et al., a small sample could be sufficient given the study’s narrow focus, the specificity of the participant group, and the detailed narratives generated [33]. Although the study aimed to achieve variation in age and gender, the final sample mainly consisted of older respondents and women, reflecting the patient population at large. However, the under-representation of younger participants and men may have limited the diversity of perspectives, affecting the transferability of our study.

## Conclusion

Self-monitoring of blood pressure was perceived as resulting in greater involvement in one’s own care and providing a better understanding of the body through self-management. Use of self-assessment questionnaires in hypertension care was described as potentially promoting deeper awareness of personal health and facilitating a collaborative patient-professional relationship. These findings suggest that sustainable self-care is contingent upon quality support and meaningful engagement between patients and healthcare professionals, underscoring that self-care in hypertension cannot be considered an entirely autonomous activity. Further research could shed light on whether self-monitoring of blood pressure combined with consistent use of self-assessment of self-care can promote a culture of collaboration and shared decision-making.

## Supplementary Information

Supplementary Material 1.

## Data Availability

No datasets were generated or analysed during the current study.
