# Supplementary material for: Self-monitoring of blood pressure and self-assessment of self-care: an interview study among patients with hypertension
Source: BMC Prim Care. 2026 Mar 14;27:125. doi: 10.1186/s12875-026-03269-7 (PMC13064247; doi:10.1186/s12875-026-03269-7)
Supplement: Supplementary file 1 — Supplementary Material 1. [file 12875_2026_3269_MOESM1_ESM.docx]

| Meaning units | Codes | Code labels | Themes |
| --- | --- | --- | --- |
| ”I know [which blood pressure] values I can accept, and if it goes beyond that, I get in touch with clinic”  ”I get nervous when I see my blood pressure increasing” | Knowing values to accept  Nervous when BP increases | Blood pressure montioring  Side-effects of BP monitoring | Understanding the body though self-management |
| “Self-assessment is a way to be more engaged in own health”  “It gives me an opportunity to think, to gain knowledge about myself, it gives an overview so I can gain more control of my life...contribute to conciousness about how I live”  “you can see that you’ve changed your thinking in health or general well-being...If someone asks how you are now, you say it's good, although you don't know what it is that is good. When you get a question on paper, you can see where things have improved” | Feeling of being more engaged  Opportunity to gain knowledge about myself  Tracking changes in health | The value of self-assessment  The value of self-assessment  The value of self-assessment | Self-assessment fosters reflection |
| ” …more information and clarity on when to change the medications”; “wish for a plan ahead”  “My body should not be a secret to me, I need to be more involved” | Wish for information;  Care expectations  Wish for involvement | Patients´care needs and expectations  Patients´care needs | Information needs and expectations to be involved |
| “When you want to make an appointment, it feels like the healthcare is dismissive, rejective and unwelcomed”  “Self-assessment questionnaire may evoke feeling of being taken more seriously” | Wish for frequent visits  Feeling of taken more seriously | Unwelcomed care experiences  The value of self-assessment | Trust through perceived engagement |
| “...good to have something to discuss about, to have someone to talk to”  “if I get viewpoints on self-assessment, I could potentially improve my health by getting advise and directions” | Self-assessment opens a dialogue  Improving health through guidance | Improved communication  Value of self-assessment through sharing it with healthcare professional | Sharing data and shared decision making |
